# 1398. Disparities in HIV continuum of care in the pediatric population: a real-life study in Brazil

**DOI:** 10.1093/ofid/ofac492.1227

**Published:** 2022-12-15

**Authors:** Alexandre A C M Ferreira, Rosana Gonçalves Gonçalves Pinho, Lais M De Aquino, Filipe B Perini, Fernanda F Fonseca, Alexsana S Tressi, Gerson F M Pereira, Vivian I Avelino-Silva, Ana Roberta Pascom

**Affiliations:** Faculdade de Medicina da Universidade de Sao Paulo, São Paulo, Sao Paulo, Brazil; Ministry of Health of Brazil, Brasília, Distrito Federal, Brazil; Ministry of Health of Brazil, Brasília, Distrito Federal, Brazil; Ministry of Health of Brazil, Brasília, Distrito Federal, Brazil; Ministry of Health of Brazil, Brasília, Distrito Federal, Brazil; Ministry of Health of Brazil, Brasília, Distrito Federal, Brazil; Ministry of Health of Brazil, Brasília, Distrito Federal, Brazil; Faculdade de Medicina da Universidade de Sao Paulo, São Paulo, Sao Paulo, Brazil; Ministry of Health of Brazil, Brasília, Distrito Federal, Brazil

## Abstract

**Background:**

The pediatric HIV follow-up is challenging, and treatment indicators are markedly distant from UNAIDS goals. In this study, we describe the 2019 Brazilian HIV cascade according to age categories and sociodemographic variables and address temporal trends between 2009-2019.

**Methods:**

We obtained data from the Brazilian Ministry of Health monitoring database. Cascade outcomes included retention in care, antiretroviral use, and viral suppression. The effect of age on timely initiation of antiretroviral treatment (ART, initiation with CD4^+^ T cell count ≥350 cells/mm^3^ or a first ART dispensation ≤30 days after the first CD4^+^ T cell measurement) and detectable HIV viral load (>50 copies/mL) was assessed in univariable and multivariable analysis adjusted for gender, race, and Social Vulnerability Index (SVI). Temporal trends in timely ART initiation and viral suppression were evaluated graphically.

**Results:**

Among 771,774 people living with HIV (PLHIV), those from youngest age categories had poorer indicators in the care cascade (Figure 1). PLHIV in younger age groups, those with higher SVI, and those declaring black and native Brazilian race/ethnicity, had higher odds of having detectable viral load and delayed ART initiation (Table 1).Social aspects are also associated with worse outcomes. Although children living with HIV tend to start ART with higher CD4^+^T cell counts, time-series analysis suggest that improvements observed in the adult population do not extend to the pediatric population (Figure 2)

Continuum of care in Brazil, 2019

**Figure:**
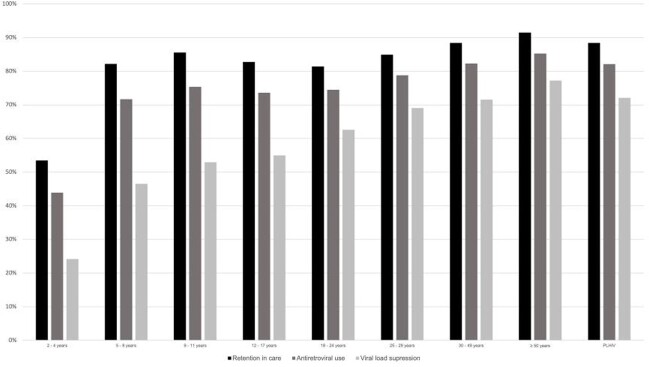

Retention in care, antiretroviral use and viral load suppression in people living with HIV under care in the Brazilian Unified Health System in 2019, overall and by age category (N=771,774).

Temporal trends, Brazil (2009-2019)

**Figure:**
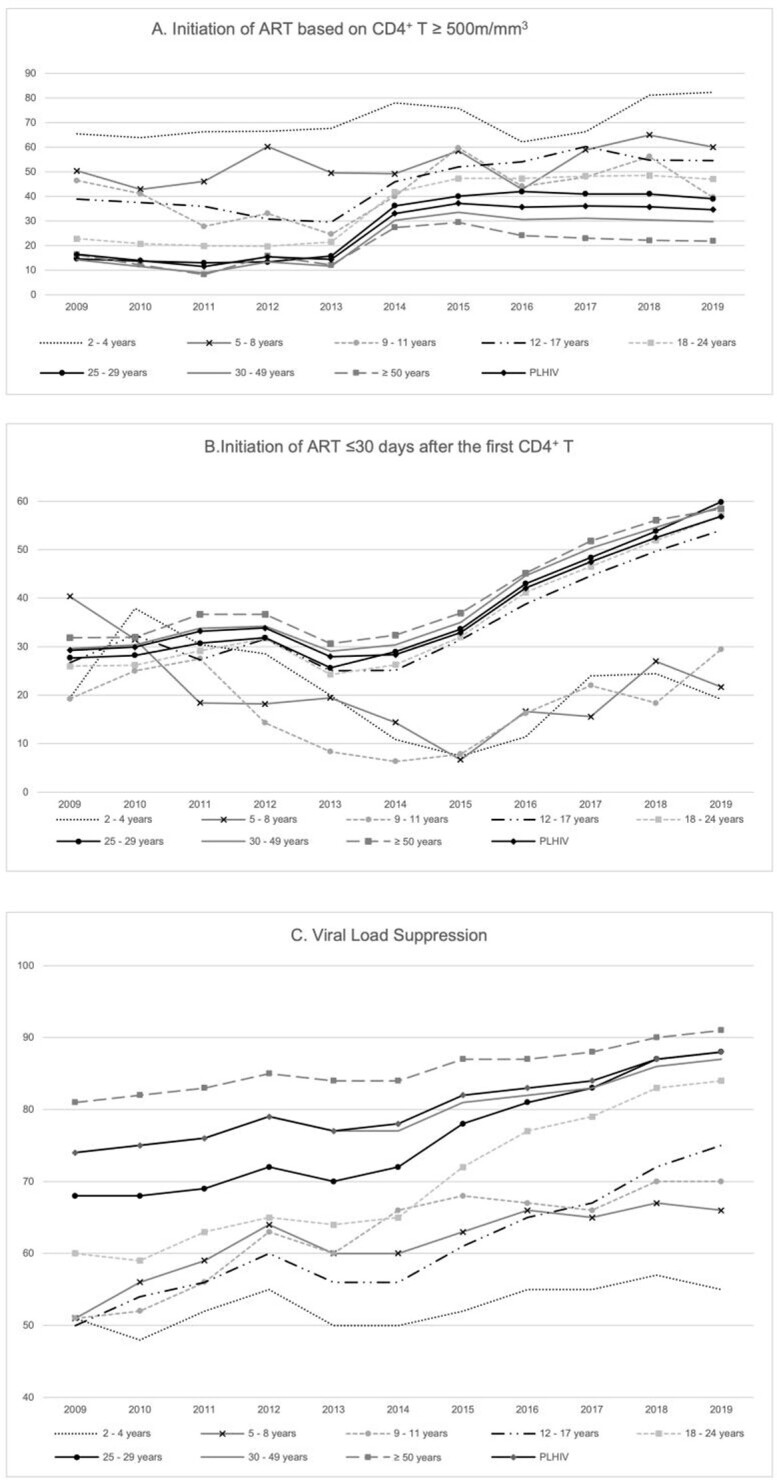

Temporal trends of timely initiation of ART based on a CD4+ T cell count ≥500m/mm3 (panel A), timely initiation of ART based on a first ART dispensation ≤30 days after the first CD4+ T cell count measurement (panel B) and viral load suppression (panel C) overall and according to age categories between 2009 and 2019 in Brazil.

**Table 1:**
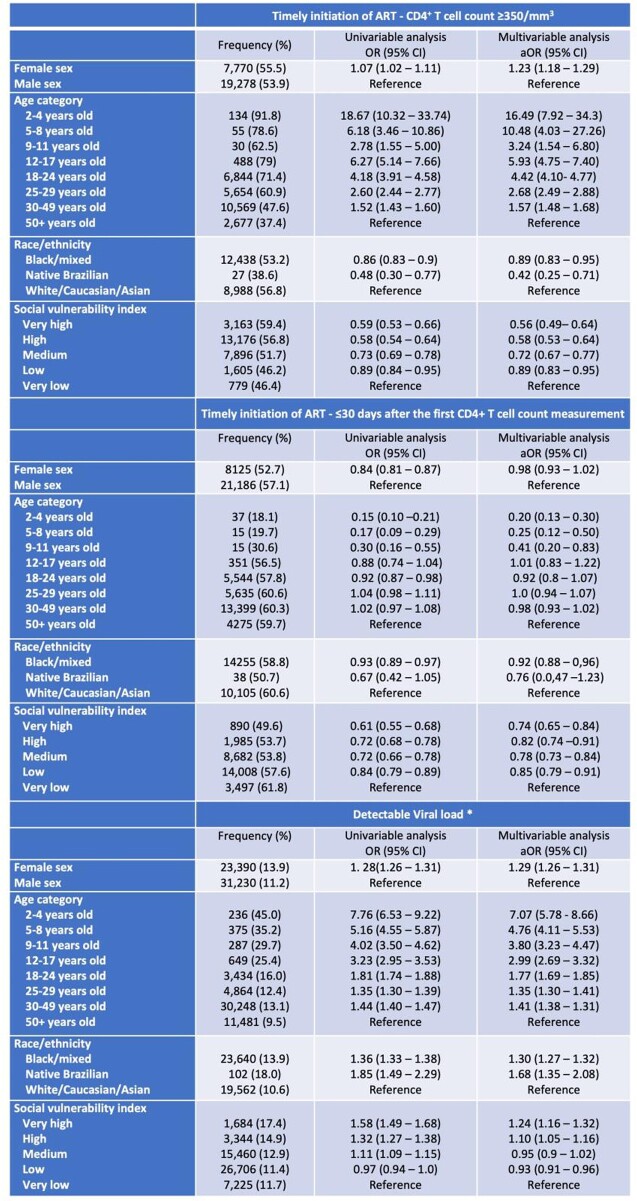
Associations between demographic variables and timely initiation of ART (based on a CD4+ T cell count ≥350m/mm3 or ≤30 days after the first CD4+ T cell count measurement) and detectable viral load using univariable and multivariable logistic regression models in the 2019 dataset.

**Conclusion:**

Our results highlight the challenge faced by children and adolescents living with HIV to achieve UNAIDS goals. Lower access to ART among children is a central barrier to improve pediatric care.

**Disclosures:**

**All Authors**: No reported disclosures.

